# Cardiovascular determinants of the 6-minute walk distance in cardiac transthyretin amyloidosis

**DOI:** 10.1186/s12872-025-05215-4

**Published:** 2025-11-14

**Authors:** Vladimir Cejka, Clemens Hosp, Maximilian Steinhardt, Aikaterini Papagianni, Sandra Ihne-Schubert, Nina Scholz, Mengmeng Chen, Julia Schäfer, Ali Adrah, Martin Kortüm, Claudia Sommer, Hermann Einsele, Stefan Frantz, Stefan Störk, Caroline Morbach

**Affiliations:** 1 Department Clinical Research & Epidemiology, University Hospital Würzburg, Comprehensive Heart Failure Center, Am Schwarzenberg 15, Würzburg, 97078 Germany; 2 Department of Medicine I, University Hospital Würzburg, Würzburg, Germany; 3 Interdisciplinary Amyloidosis Center Northern Bavaria, Würzburg, Germany; 4 Department of Medicine II, University Hospital Würzburg, Würzburg, Germany; 5 Department of Neurology, University Hospital Würzburg, Würzburg, Germany; 6 Department of Internal Medicine IV, University Hospital Gießen and Marburg, Gießen, Germany; 7 CIRCLE - Centre for Innovation Research, Lund University, Lund, Sweden

**Keywords:** Cardiac transthyretin amyloidosis, ATTR, Cardiomyopathy, 6-minute walk distance, Functional capacity

## Abstract

**Background:**

The six-minute walk distance (6MWD) is a measure of functional capacity and a frequently used endpoint in clinical trials investigating transthyretin amyloid cardiomyopathy (ATTR-CM). We evaluated the clinical utility of the 6MWD by quantifying the gap between the expected and observed physical performance and estimating its determinants.

**Methods:**

Outpatients with wild-type (ATTRwt)-CM were investigated. Standardized echocardiographic, laboratory and clinical assessments were performed. A regression formula derived from a healthy local population sample was applied to predict the expected 6MWD. Associations with 6MWD were analyzed by linear regression, adjusted for age and height. Explanatory multivariable models using backward elimination, regularization and clinical reasoning were calculated.

**Results:**

100 patients were analyzed. Their mean age was 78.7 (6.3) years and 86% were men. The mean observed 6MWD was 310 (113) m, which corresponded to about 65% of the expected performance. Significant predictors of the 6MWD were (ordered by decreasing explanatory power): high-sensitivity Troponin T, NT-proBNP, NAC disease stage, estimated glomerular filtration rate, atrial fibrillation, hemoglobin, hepatic vein dilation, mitral E-wave maximum velocity, left ventricular ejection fraction, and the tricuspid valve maximal regurgitation pressure gradient. Multivariable models yielded an R² of up to 45.9% with a root mean squared error of 82.9 m.

**Conclusions:**

Physical performance as measured by the 6MWD in patients with ATTRwt-CM was remarkably compromised. Laboratory and imaging markers indicative of disease severity and congestion predicted the 6MWD in these patients. Cardiovascular markers explained a fair amount of 6MWD variability.

**Supplementary Information:**

The online version contains supplementary material available at 10.1186/s12872-025-05215-4.

## Introduction

When transthyretin (ATTR) amyloidosis starts to affect the heart, the resulting cardiomyopathy (ATTR-CM) almost inevitably has grave implications on an individual´s quality of life (QoL), physical performance, and prognosis [1]. The prevalence of wild type (ATTRwt)-CM is quickly rising due to better diagnostic modalities in an aging population, whereas CM in the hereditary type (ATTRv) is less common [1, 2]. It has been estimated that eight million persons world-wide might be affected by ATTRwt-CM, and this number is projected to increase up to 26 million by the year 2050 [3, 4]. Up to recently, ATTR-CM was an underappreciated condition, as treatment was restricted to fluid management (and liver transplantation in ATTRv). New therapeutic options, however, were shown to slow progression and improve survival [5, 6]. 

ATTR-CM is an important cause of heart failure and specifically acknowledged in current treatment guidelines [7]. In the management of heart failure, medication can not only prolong survival and reduce hospitalizations, but also maintain functional capacity [7]. The distance a person can walk within six minutes (6MWD) is a validated measure of functional capacity and a frequently used endpoint in clinical trials including patients with heart failure and/or cardiac amyloidosis [5, 7–9]. The 6MWD correlates well with QoL and frailty, i.e., multi-domain constructs that are not easy to obtain in clinical practice regularly, yet essential for the comprehensive assessment of a patient´s health status [10–13]. Moreover, the 6MWD has been shown to predict death and cardiovascular hospitalizations in patients with ATTR-CM [1, 14–17].

While there is agreement on the clinical importance of the 6MWD, little is known about its determinants. Studies in healthy adults yielded age, sex, height, or weight as relevant determinants [18, 19]. In patients with heart failure, diabetes mellitus, kidney function, and natriuretic peptides predicted the 6MWD [18, 19]. However, the determinants of the 6MWD in ATTR-CM are still subject to research. In the ATTR-ACT trial, treatment with tafamidis was associated with better prognosis compared to placebo, yet a decline in the 6MWD was observed in both treatment arms. Further, in patients treated with tafamidis, levels of natriuretic peptides remained unaffected and no reverse left ventricular remodeling could be observed [5]. In contrast, post-hoc analyses from trials applying gene silencers to patients with ATTRv-CM showed favorable effects on functional capacity, cardiac enzymes, and echocardiographic parameters [20–23]. Nevertheless, restoration of euvolemia and optimization of heart failure pharmacotherapy in ATTR-CM most likely also impacts physical capacity – independent of amyloid disease progression.

To appreciate the utility of the 6MWD as a clinical measure and valuable endpoint surrogate in this fragile group of patients, we (a) contrasted the 6MWD of ATTRwt-CM patients with the 6MWD predicted by a regression formula derived from a healthy local population sample, (b) studied the association of the 6MWD with anthropometric variables, clinical characteristics including cardiac structure and function, and medication, (c) developed explanatory models of the 6MWD using backward elimination, regularization and clinical reasoning guided regression techniques.

## Methods

### Study population

This cross-sectional analysis included consecutive outpatients with confirmed ATTRwt-CM, who had been referred to the Interdisciplinary Amyloidosis Center of Northern Bavaria between November 2018 and December 2021. The Ethics Committee of the Medical Faculty Würzburg, Germany, waived informed consent regarding this retrospective collection and analysis of clinical data (waiver #20211011 02). Outpatient visits were performed at the Comprehensive Heart Failure Center (CHFC) Würzburg, which aims to integrate patient care and clinical research. Therefore, all investigations are carried out according to dedicated standard operating procedures by specifically trained staff. The diagnosis of cardiac ATTRwt-CM was ascertained either (i) by immunohistochemical detection of ATTR amyloid fibril protein deposition in myocardial biopsy, or (ii) by a positive bone scintigraphy scan (applying DPD or DPD equivalent) Perugini grade 2 or 3 [24] in the absence of monoclonal gammopathy (operationalized as a normal free light chain ratio, negative immune fixation of the serum, negative immune fixation of 24-hour collected urine, and absent M-gradient in serum electrophoresis). Rule-out of monoclonal gammopathy and hematologic tests were supervised by an expert hematologist [6]. All patients received genetic testing, including sequencing of the transthyretin gene.

### Clinical assessment

At the day of the visit at our center, all patients received a comprehensive work-up. Height was measured standing supine with a stadiometer in cm. Weight was measured using calibrated scales, with the person in underwear and shoes taken off. Diabetes mellitus (DM) was diagnosed in patients with a history of DM, HbA1c >6.5%, or on antidiabetic medication. Echocardiography was performed according to pre-specified standards including parasternal, apical and subcostal views using a Vivid E9 or Vivid E95 machine (GE Healthcare, Horten, Norway). Dimensions, systolic and diastolic functional parameters, as well as valvular disease were assessed according to current recommendations and included the recording of left ventricular (LV) outflow tract diameter (parasternal long axis) and pulsed-wave Doppler (apical five chamber view) to calculate LV stroke volume and cardiac output as well as apical four-, two-, and three chamber views to determine LV ejection fraction (LVEF, Simpson´s biplane method) and global longitudinal strain. Hepatic veins were visualized from the subcostal angle of view. The width of the hepatic veins was measured ≥ 2 cm proximal of the ostium of the vena cava inferior. Hepatic vein dilation was defined as width >10 mm in ≥ 1 hepatic vein. ATTRwt-CM was classified into three NAC (National Amyloidosis Center in London, UK) disease stages (I/II/III), whereby higher stages indicated more advanced disease. Stage I was defined as N-terminal pro-hormone B-type natriuretic peptide (NT-proBNP) ≤ 3000 pg/ml and estimated glomerular filtration rate (eGFR) ≥ 45 ml/min/1.73 m²; stage II was defined as either NT-proBNP >3000 pg/ml or eGFR < 45 ml/min/1.73 m²; stage III was defined as NT-proBNP >3000 pg/ml and eGFR < 45 ml/min/1.73 m² [25]. Venous blood was drawn under fasting conditions and analyzed in the Central Laboratory of the University Hospital Würzburg, Germany. Creatinine was measured by the enzymatic method with the Roche-Cobas assay, NT-proBNP with the Roche-Cobas immunoassay, and high-sensitivity (hs) Troponin T with the Cobas e 601 and Cobas e 411 immunoassays. The eGFR was estimated by the CKD-EPI equation [26].

### Six-minute walk test in patients with ATTRwt-CM and healthy controls

We performed the six-minute walk test (6MWT) in an undisturbed, straight and flat indoor hallway with a marked test walk of 15 m length. Each participant without contraindications (e.g., instable angina pectoris or myocardial infarction within the previous four weeks, blood pressure > 180/100 mmHg, or resting heart rate > 120 bpm) completed the 6MWT under the supervision of a trained staff member. Patients were instructed to cover as much ground as possible within six minutes, without running or jogging. They could slow down or stop if necessary, but should resume walking as soon as possible. The distance walked was calculated from the number of 15 m laps completed within the six minutes plus the remaining meters of the last, incomplete lap. Heart rate was measured manually in standing position immediately before and after the 6-minute walk by an experienced nurse.

To enable the comparison between the physical capacity of patients and otherwise healthy individuals, we made use of data collected with an identical 6MWT protocol from a large local population-based sample of the Characteristics and Course of Heart Failure Stages A-B and Determinants of Progression (STAAB) Cohort Study. STAAB recruited a representative sample of 4965 residents of the City of Würzburg, Germany, who were aged between 30 and 80 years at study start and were free from heart failure. Detailed design and characteristics of the STAAB study have been published [27, 28]. 2762 participants attending the first follow-up examination provided a valid 6MWT. Out of those, a subgroup of 681 individuals free from cardiovascular disease and cardiovascular risk factors was deemed “apparently healthy”. From this subgroup, normal reference values were derived and published [29]. Age and height were found to be independent predictors of the 6MWD in the general population, whereas sex was not [29]. 

### Data analysis 

To quantify the difference between the observed 6MWD and a ‘healthy’ 6MWD, we used the 6MWD prediction formula derived from the STAAB study as a reference [27, 29]. We calculated the predicted (healthy) 6MWD for each participant from the respective linear regression formula as follows:

$$\begin{aligned}&\mathrm{Predicted}\;6\mathrm{MWD}\;(\mathrm m)=592.134+0.203\\&\ast\left(\mathrm{Age}\left[\mathrm{years}\right]<56.2\right)\\&\ast\left(56.2-\mathrm{age}\left[\mathrm{years}\right]\right)-5.034\\&\ast\left(\mathrm{age}\left[\mathrm{years}\right]>56.2\right)\\&\ast\left(\mathrm{age}\left[\mathrm{years}\right]-56.2\right)+1.857\\&\ast\left(\mathrm{height}\left[\mathrm{cm}\right]-172.6\right)\end{aligned}$$

The difference between the observed and predicted 6MWD was calculated for each participant by subtracting the predicted 6MWD from the observed 6MWD. The percent of predicted 6MWD was calculated for each participant by dividing the observed 6MWD by the predicted 6MWD.

We explored clinical, echocardiographic and laboratory parameters as well as medication as candidate predictors of the observed 6MWD (for the full list of variables, refer to Table 2). Body Mass Index (BMI) was calculated as weight in kg divided by height squared in m. All analyzed variables were assessed for normality by histograms and Q-Q plots. Natural log-transformation for linear regression modelling was performed for non-normally distributed variables to better approximate a normal distribution. Descriptive statistics of the study sample are given as number (percent), mean (standard deviation) or median (quartiles) according to the nature of the data. Group comparisons were done using Student´s t-test, Mann-Whitney U-test and chi-square test, as appropriate. Atrial fibrillation, NYHA functional class, medications, hepatic vein congestion, disease stage and sex were analyzed as categorical variables, all other variables were analyzed as continuous variables. Determinants of the observed 6MWD were analyzed in multiple steps by linear regression modelling. First, to assess the impact of basic patient characteristics, the effect of age, height, sex and BMI on the observed 6MWD was examined in separate univariable models. Second, for each further candidate predictor analyzed, an adjusted linear regression model was constructed using the variables selected in the first step (based on model performance characteristics as p-value, effect size, explained variance) as fixed factors, while further adding the candidate predictor (variable of interest). See Supplementary Figure S1 for more detail on univariable and adjusted analysis. This approach yielded trivariable, age- and height-adjusted, linear regression models of candidate predictors. Standardized beta coefficients were computed to compare the adjusted effect sizes across predictors. To integrate the information from all trivariable models calculated, we aimed to build an explanatory multivariable 6MWD linear regression model using age, height, and significant predictors identified in trivariable regression models. For this purpose, three multivariable modeling approaches with the observed 6MWD as the dependent were undertaken [30]. We focused on model parsimony because of limited power due to the sample size of 100 participants. In order to harness the full sample and avoid case-wise deletion in multivariable regression modelling, the few missing data points were imputed by their corresponding variable median. The first multivariable model was built by Akaike Information Criterion [31] (AIC) based backward elimination stepwise regression, where the initial linear regression model included age and height as fixed predictors and all significant predictors identified in trivariable modeling. Age and height were fixed because they were considered reliable determinants of the 6MWD based on prior research [32] and univariable analyses in this study (Supplementary Figure S1). The stepwise approach iteratively eliminates predictors and compares models until the model with the lowest AIC, which balances model likelihood and the number of predictors, is reached. The next multivariable model was built by Least Absolute Shrinkage and Selection Operator (LASSO) linear regression modelling. Age and sex were kept as fixed predictors as explained above. The LASSO modeling approach applies a penalty to all regression coefficients (except the fixed coefficients of age and sex). The penalty factor λ (lambda) eventually shrinks some coefficients to zero, thus eliminating predictor variables. A full model including age, sex and all significant trivariable predictors was built. To find the optimal shrinkage factor, we used nested 5-fold cross validation [33]. The dataset was randomly split 5-times by an 80/20 ratio into a training and testing set. The optimal λ from the training sets (found by 5-fold cross validation) were than applied to the test sets. The λ for the final LASSO linear regression model was chosen based on the lowest root mean squared error (RMSE) in the test set. Because the LASSO procedure (which yielded the final LASSO model) does not provide unbiased regression estimates and standard errors, a post-LASSO ordinary least squares linear regression model was fit using the predictors identified by the final LASSO model. In contrast to backward elimination and LASSO, both algorithmic approaches, we built also a multivariable model based on clinically guided reasoning and evaluation of significant trivariable model predictors with respect to variance explained as measured by R². Briefly, predictors were added stepwise into a multivariable linear regression model, in decreasing order of the variance that they explained in trivariable (age- and height-adjusted) models. Predictors that reached statistical significance expanded the previous model in a stepwise manner. A full description and rationale of this modelling approach is provided in the Supplementary Comment and Figure S2.

A p-value < 0.05 was considered statistically significant. R² values presented are unadjusted. As all reported associations are considered exploratory, no multiple-testing corrections were applied. Calculations were performed in IBM SPSS Statistics, Version 28, and R (R Core Team (2023) and R: A Language and Environment for Statistical Computing. R Foundation for Statistical Computing, Vienna, Austria, URL https://www.R-project.org/) using the packages caret, glmnet and Metrics.

## Results

We included 100 patients with ATTRwt-CM into the present analysis. The number of missing data was generally low (maximum 4% per variable). The mean age was 78.7 (standard deviation: 6.3) years, and 86 (86%) were men. The mean observed 6MWD was 310 (113) m, and was 317 (115) m for men and 267 (97) m for women. The mean predicted 6MWD was 475 (40) m, corresponding to mean performance of 65 (40) % of the predicted 6MWD. The respective performance for men was 66 (23) % and was 60 (21) % for women. The mean body mass index was 27.4 (3.4) kg/m², 19% had diabetes mellitus and 52% had atrial fibrillation. The NAC (National Amyloidosis Center London, United Kingdom) disease stage I/II/III proportion according to Gillmore et al. [25] was 44%/33%/23%, respectively, and 55% were in New York Heart Association functional class III. The median levels for eGFR were 48 (37; 63) ml/min/1.73 m², for NT-proBNP 2880 (1554; 4857) pg/ml, and for hs-Troponin T 46 (34; 70) pg/ml. Mean left ventricular ejection fraction was 53 (10) %, interventricular septum thickness 19 (3) mm, and 53% were treated with tafamidis. Descriptive statistics of the total study sample and subgroups split at the median age of 80 years are summarized in Table 1.

**Table 1:** Patient characteristics

Variable (Unit)	Total sample (*N* = 100)	Subjects aged < 80 years (*n* = 50)	Subjects aged ≥ 80 years (*n* = 50)	*P*- value	
Age (years)	78.7 (6.3)	73.8 (4.5)	83.6 (3.2)	-	
Male sex, N (%)	86 (86)	45 (90)	41 (82)	0.388	
Height (m)	1.71 (0.08)	1.73 (0.08)	1.68 (0.08)	0.003	
Body mass index (kg/m²)	27.5 (3.4)	28.5 (3.3)	26.5 (3.2)	0.002	
Systolic blood pressure (mmHg)	137 (20)	138 (20)	136 (20)	0.639	
Diastolic blood pressure (mmHg)	81 (13)	82 (13)	80 (13)	0.342	
Heart rate (beats/min)	70 (14)	64 (11)	70 (16)	0.022	
Difference in heart rate before and after 6MWT (beats/min)	20 (12; 31)	19 (12; 29)	21 (13; 31)	0.631	
**Comorbidity and symptom burden**					
NAC disease stage, N (%) [25]				0.380	
I	44 (44)	25 (50)	19 (38)		
II	33 (33)	16 (32)	17 (34)		
III	23 (23)	9 (18)	14 (28)		
Diabetes mellitus, N (%)	19 (19)	8 (16)	11 (22)	0.611	
Atrial fibrillation, N (%)	52 (52)	26 (52)	26 (52)	> 0.99	
Pacemaker, N (%)	18 (18)	9 (18)	9 (18)	> 0.99	
NYHA functional class, N (%)				0.192	
I	8 (8)	5 (10)	3 (6)		
II	37 (37)	22 (44)	15 (30)		
III	55 (55)	23 (46)	32 (6)		
**Physical capacity (6MWD)**					
Observed 6MWD (m)	310 (113)	346 (91)	274 (122)	0.001	
Predicted 6MWD (m)	475 (40)	505 (30)	446 (23)	< 0.001	
Difference observed vs. predicted 6MWD (m)	−165 (102)	−158 (91)	−172 (112)	0.486	
Observed 6MWD as percent of predicted 6MWD (%)	65 (22)	69 (18)	61 (26)	0.078	
**Pharmacotherapy**					
Tafamidis, N (%)	53 (53)	29 (58)	24 (48)	0.423	
Betablocker, N (%)	59 (59)	31 (62)	28 (56)	0.685	
ARNI, N (%)	8 (8)	6 (12)	2 (4)	0.269	
ACE-i or ARB, N (%)	50 (50)	23 (46)	27 (54)	0.549	
MRA, N (%)	32 (32)	12 (24)	20 (40)	0.133	
Diuretic, N (%)	86 (86)	39 (78)	47 (94)	0.041	
**Echocardiography**					
Left ventricular ejection fraction (%)	53 (10)	53 (10)	53 (10)	0.960	
MAPSE septal (mm)	6.0 (3.0; 8.0)	7.0 (5.0; 9.0)	6.0 (5.0; 8.0)	0.641	
MAPSE lateral (mm)	7.0 (6.0; 10.0)	7.0 (6.0; 10.0)	8.0 (5.5; 9.5)	0.135	
Global longitudinal peak strain (%)	−10.7 (−12.9; −8.0)	−10.7 (−12.8; −8.0)	−10.8 (−13.1; −8.1)	0.825	
Left ventricular s´ wave (ms)	0.042 (0.013)	0.045 (0.013)	0.039 (0.012)	0.024	
Right ventricular s´ wave(ms)	0.10 (0.03)	0.10 (0.03)	0.10 (0.03)	0.451	
IVSD (mm)	19 (3)	19 (4)	18 (3)	0.166	
LPWD (mm)	14 (2)	15 (2)	15 (2)	0.928	
LVEDV (ml)	100 (78; 127)	112.5 (96; 133)	82 (70; 113)	< 0.001	
Left atrial volume (ml)	96 (78; 113)	99 (87; 113)	93 (74; 112)	0.129	
E wave (m/s)	0.88 (0.22)	0.87 (0.19)	0.9 (0.24)	0.496	
E/E´	18 (15; 24)	18 (13; 21)	19 (15; 24)	0.135	
E´ wave septal (m/s)	0.04 (0.01)	0.05 (0.02)	0.04 (0.01)	0.026	
E´ wave lateral (m/s)	0.06 (0.03)	0.06 (0.03)	0.06 (0.04)	0.515	
Stroke volume (ml)	71 (19)	76 (20)	67 (19)	0.027	
Cardiac output (l/min)	4.55 (3.87; 5.5)	4.53 (3.92; 5.60)	4.55 (3.78; 5.13)	0.393	
TAPSE (mm)	15 (12; 19)	16 (12; 20)	15 (12; 18)	0.315	
Hepatic vein dilation, N (%)	21 (21)	6 (12)	15 (30)	0.028	
TR-Pmax (mmHg)	36 (30; 46)	36 (29; 43)	36 (32; 46)	0.184	
**Laboratory parameters**					
eGFR (ml/ml/1.73 m²)	48 (35; 63)	53 (34; 65)	46 (36; 53)	0.054	
NT-proBNP (pg/ml)	2880 (1554; 4857)	2343 (1166; 3937)	3511 (2130; 5830)	0.003	
High-sensitivity Troponin T (pg/ml)	46 (34; 70)	44 (30; 65)	50 (36; 80)	0.054	
Hemoglobin (g/dl)	13.4 (1.8)	13.5 (1.8)	13.2 (1.9)	0.498	

Data are mean (SD) or median (quartiles), unless indicated otherwise. P-values are from t-test, Mann-Whitney U-test, or chi-square test, as appropriate. 6MWD: 6-minute walk distance. *ACE-i* angiotensin converting enzyme inhibitor, *ARB* angiotensin receptor blocker, *ARNI* angiotensin receptor-neprilysin inhibitor, Diuretic: loop, thiazide or thiazide-like diuretic, *eGFR* estimated glomerular filtration rate, *IVSD* interventricular septum diameter, *LPWD* left ventricular posterior wall diameter, *LVEDV* left ventricular end-diastolic volume, *MAPSE* mitral annular plane systolic excursion, *MRA* mineralocorticoid receptor antagonist,*NT-proBNP* amino-terminal pro-hormone natriuretic peptide type B, *NYHA* New York Heart Association, *TAPSE* tricuspid annular plane systolic excursion, *TR-Pmax* tricuspid regurgitation maximal pressure

In univariable analysis, age: beta − 7.7 (95% CI: −11.0; −4.5, *p* < 0.001) m per year, and height: beta − 3.45 (95% CI: −0.81; −6.10, *p* = 0.011) m per cm, but not sex or body mass index (both *p* > 0.05), were significant predictors of the 6MWD (Supplementary Figure S1). The R² of the linear regression model including age and height as independent variables was 0.196. 

**Fig. 1:**
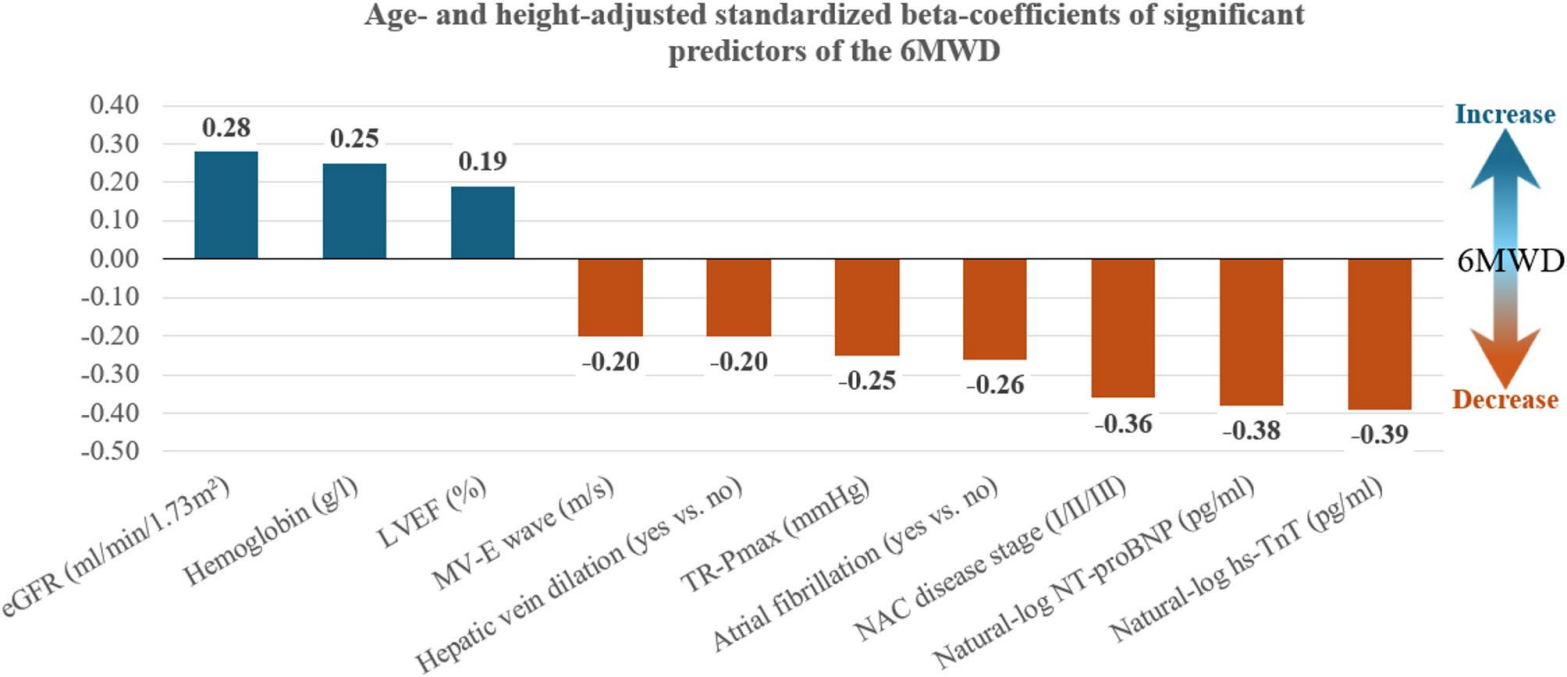
Significant standardized age- and height-adjusted beta regression coefficients of the 6MWD. Legend: Bar plot showing the significant age- and height-adjusted standardized beta-coefficients from linear regression analyses. The x-axis includes a bar for each predictor. The y-axis represents the magnitude of the effect size (standardized beta). For each predictor, a separate trivariable model, adjusted for age and height, was created. Blue bars indicate an increase in 6MWD with increasing effect size. Orange bars indicate a decrease in 6MWD with increasing effect size. Atrial fibrillation and hepatic vein congestion as nominal variables, and NAC disease stage as ordinal variable. 6MWD: 6-minute walk distance, eGFR: estimated glomerular filtration rate, LVEF: left ventricular ejection fraction, MV-E wave: mitral valve E wave, NT-proBNP: N-terminal pro-natriuretic peptide type B, hs-TnT: high sensitivity Troponin T, TR-Pmax: tricuspid regurgitation maximal pressure

Trivariable (age- and height-adjusted) linear regression models of candidate predictors adjusted for age and height identified the following further significant correlates of the 6MWD in patients with ATTRwt-CM (ordered by decreasing R²) - hs-Troponin T: beta − 77 (95% CI: −111; −43, *p* < 0.001) m per natural log-pg/ml, NT-proBNP: beta − 51 (95% CI: −75; −27, *p* < 0.001) m per natural log-pg/ml, NAC disease stage: beta − 51 (95% CI: −76; −26, *p* < 0.001) m per one stage increase, eGFR: beta:1.91 95% CI: (0.69; 3.13, *p* = 0.002) per ml/min/1.73 m², atrial fibrillation: beta − 59 (95% CI: −98; −19, *p* = 0.004) m if present, hemoglobin: 15.6 (95% CI: 4.5; 26.8, *p* = 0.006) m per g/dl, hepatic vein dilation: beta − 55 (95% CI: −106; −4, *p* = 0.034) m if present, mitral E-wave maximum velocity: beta − 101 (95% CI: −194; −88, *p* = 0.034) m per m/s, LVEF: beta 2.13 (95% CI: 0.15; 4.11, *p* = 0.035) m per %, and tricuspid regurgitation maximal pressure gradient (TR-Pmax): beta − 2.76 (95% CI: −4.80; −0.71, *p* = 0.009) m per mmHg. The explained variance of the 6MWD went up to 32 and 33% for NT-proBNP and hs-Troponin T, respectively. The effects of all other analyzed variables, including medication, did not reach statistical significance and did not increase materially the respective variance explained by the trivariable models. These results are summarized in Table 2. When comparing the effect sizes of significant trivariable predictors, hs-Troponin T exhibited the strongest age- and height-adjusted effect, followed by NT-proBNP and NAC disease stage. The standardized effects of significant trivariable predictors are shown in Fig. 1.

**Table 2:** Determinants of the 6MWD (age- and height-adjusted linear regression analyses)

Parameter (unit)	Beta-coefficient (95% confidence interval)	*P*-value	*R*²
Echocardiography			
**Left ventricular ejection fraction (%)**	**2.13 (0.15; 4.11)**	**0.035**	**0.233**
Global longitudinal peak strain (%)	−4.33 (−9.31; 0.66)	0.088	0.219
MAPSE septal (mm)	2.40 (−4.43; 9.22)	0.488	0.200
MAPSE lateral (mm)	7.29 (−0.23; 14.81)	0.057	0.226
**Mitral valve E wave velocity (m/s)**	**−101 (−194; −88)**	**0.034**	**0.233**
Mitral E/E´ average	−1.83 (−4.55; 0.89)	0.185	0.211
Mitral E´ wave velocity septal (m/s)	457 (−1079; 1994)	0.556	0.199
Mitral E´ wave velocity lateral (m/s)	−187 (−801; 4266)	0.545	0.199
IVSD (mm)	−0.65 (−7.20; 5.89)	0.843	0.197
LPWD (mm)	−5.35 (−14.32; 4.29)	0.255	0.207
LVEDV (ml)	−0.47 (−1.13; 0.20)	0.166	0.212
Left atrial volume (ml)	−0.57 (−1.34; 0.21)	0.150	0.214
Cardiac output (l/min)	13.8 (−1.6; 29.29)	0.078	0.227
Stroke volume (ml)	0.19 (−0.92; 1.30)	0.729	0.202
TAPSE (mm)	4.03 (−0.19; 8.26)	0.061	0.225
Right ventricular s´ wave (m/s)	474 (−199; 1148)	0.165	0.212
**TR-Pmax (mmHg)**	**−2.76 (−4.80; −0.71)**	**0.009**	**0.232**
**Hepatic vein dilation (yes vs. no)**	**−55 (−106; −4)**	**0.034**	**0.240**
Laboratory parameters			
**Ln NT-proBNP (pg/ml)**	**−51 (−75; −27)**	**< 0.001**	**0.322**
**Ln hs-TnT (pg/ml)**	**−77 (−111 −43)**	**< 0.001**	**0.335**
**eGFR (ml/min/1.73 m²)**	**1.91 (0.69; 3.13)**	**0.002**	**0.270**
**Hemoglobin (g/dl)**	**15.6 (4.5; 26.8)**	**0.006**	**0.257**
Pharmacotherapy			
Betablocker (yes vs. no)	1.48 (−40.72; 43.67)	0.945	0.196
ACE-inhibitor (yes vs. no)	26.56 (−20.00; 73.13)	0.260	0.207
ARB (yes vs. no)	−16.68 (−65.28; 31.92)	0.497	0.200
ARNI (yes vs. no)	27.52 (−49.49; 104.54)	0.480	0.200
MRA (yes vs. no)	9.23 (−34.77; 53.22)	0.678	0.198
Diuretic (yes vs. no)	6.37 (−50.30; 63.04)	0.856	0.197
Tafamidis (yes vs. no)	−11.99 (−53.21; 29.23)	0.565	0.199
Other cardiovascular factors			
**Atrial fibrillation (yes vs. no)**	**−59 (−98; −19)**	**0.004**	**0.267**
Pacemaker (yes vs. no)	−8.3 (−61.9; 45.2)	0.758	0.197
**NAC disease stage (I/II/III)**	**−51 (−76; −26)**	**< 0.001**	**0.315**
Heart rate before 6MWT (beats/min)	0.36 (−1.17; 1.94)	0.623	0.198
Heart rate after 6MWT (beats/min)	0.93 (−0.19; 2.06)	0.104	0.212
Difference in heart rate before and after 6MWT (beats/min)	0.90 (−0.45; 2.25)	0.189	0.204

Variables in bold print indicate significant effects.

*R²* explained variance, *ACE* angiotensin converting enzyme, *ARB* angiotensin receptor blocker, *ARNI* angiotensin receptor-neprilysin inhibitor, Diuretic: loop, thiazide or thiazide-like diuretic, *eGFR* estimated glomerular filtration rate according to the CKD-EPI formula, *IVSD* interventricular septum diameter, *LPWD* left ventricular posterior wall diameter, *LVEDV* left ventricular end-diastolic volume, *MAPSE* mitral annular plane systolic excursion, *MRA* mineralocorticoid receptor antagonist, Mitral E/E´ average: mean fomr mitral E/E´ septal and mitral E/E´ lateral, *NAC* National Amyloidosis Center London, *NT-proBNP* amino-terminal pro-hormone natriuretic peptide type B, *TAPSE* tricuspid annular plane systolic excursion, hs TnT: high-sensitivity Troponin T, TR-Pmax: tricuspid regurgitation maximal pressure

Multivariable linear regression modelling using AIC based backward elimination stepwise regression included the following predictors beyond age height (which were fixed covariates in the model): mitral E-wave maximum velocity, TR-Pmax, hs-Troponin T (natural log), atrial fibrillation and NAC disease stage. Of those, significant predictors were mitral E-wave maximum velocity: beta − 85 (−167; −2) m per m/s and hs-Troponin T: beta − 77 (−85, −8) m per natural log-pg/ml. This model yielded an R² of 0.443 and a RMSE of 84.14 m. Multivariable linear regression modelling using LASSO regression included the following predictors beyond age and height: LVEF, mitral E-wave maximum velocity, TR-Pmax, hs-Troponin T (natural log), hemoglobin, atrial fibrillation and NAC disease stage. Final LASSO regression derived beta coefficients, model R² and RMSE are provided in Supplementary Table S3. In post-LASSO ordinary least squares regression including these variables, none of them reached statistical significance. The post-LASSO ordinary least squares regression model yielded an R² of 0.459 and a RMSE of 82.90 m. Multivariable linear regression modelling using clinical reasoning included the following predictors beyond age and height: hs-Troponin T (natural log), NAC disease stage, atrial fibrillation and mitral E-wave maximum velocity. Of those, significant predictors were mitral E-wave maximum velocity: beta − 94 (−176; −12) m per m/s, hs-Troponin T: beta − 50 (−89, −11) m per natural log-pg/ml and atrial fibrillation: beta − 40 (−76; −4) m if present. The clinical model yielded an R² of 0.431 and a RMSE of 85.10 m. A summary of the multivariable models is given in Table 3. All three multivariable modelling approaches included beyond age and height the following predictors: mitral valve E wave velocity, high-sensitivity Troponin T, atrial fibrillation, NAC disease stage.

**Table 3:** Multivariable models

	AIC backward elimination model		Post-LASSO model		Clinical model	
**R²**	0.443		0.459		0.431	
**RMSE (m)**	84.14		82.90		85.05	
**Predictor**	**Beta (95% CI)**	**P-value**	**Beta (95% CI)**	**P-value**	**Beta (95% CI)**	**P-value**
Age (yrs)	−5.03 (−8.15; −1.92)	0.002	−5.20 (−8.31; −2.09)	0.001	−4.98 (−8.12; −1.85)	0.002
Height (m)	1.27 (−1.08; 3.62)	0.285	1.00 (−1.38; 3.38)	0.407	1.46 (−0.88; 3.81)	0.218
Mitral valve E wave velocity (m/s)	−84.73 (−167.49; −1.97)	0.045	−83.04 (−166.46; 0.39)	0.051	−93.78 (−175.99; −11.57)	0.026
High-sensitivity Troponin T (natural log-pg/ml)	−46.48 (−85.31; −7.64)	0.020	−36.06 (−76.83; 4.71)	0.082	−50.12(−88.82; −11.42)	0.012
Atrial fibrillation (yes vs. no)	−35.61 (−71.87; 0.65)	0.054	−28.61 (−65.71; 8.50)	0.129	−39.64 (−75.64; −3.63)	0.031
NAC disease stage (I/II/III)	−26.69 (−54.39; 1.02)	0.059	−24.68 (−52.39; 3.04)	0.080	−26.94 (−54.79; 0.90)	0.058
TR-Pmax (mmHg)	−1.34 (−3.21; 0.54)	0.160	−1.47 (−3.37; 0.42)	0.126		
Left ventricular ejection fraction (%)			1.36 (−0.51; 3.23)	0.153		
Hemoglobin (g/dl)			5.86 (−4.99; 16.71)	0.286		

Beta: beta regression coefficient *CI* confidence interval, *eGFR* estimated glomerular filtration rate according to the CKD-EPI formula, *NAC* National Amyloidosis Center London, *RMSE* root mean squared error, *TR-Pmax* tricuspid regurgitation maximal pressure

## Discussion

The current study investigated an extensively characterized convenience sample of consecutive ambulatory patients with ATTRwt-CM and reports three main findings. First, compared to age- and height-matched healthy controls, physical performance as measured by the 6MWD in patients with ATTRwt-CM was heavily compromised. Second, the identified determinants of the 6MWD were laboratory and imaging markers indicative of disease progression and congestion in these patients. Third, multivariable regression modelling suggests that age, height and the cardiovascular markers investigated here explain a considerable amount of 6MWD variance, yet they do not offer enough predictive accuracy to be decisively used in clinical practice.

We consecutively recruited patients admitted to our center and treated in real world practice. The thus generated convenience sample had a mean age of 79 years and the predominance of men (86%) closely resembled other cohorts with ATTR-CM [1, 6, 15, 20, 25]. There are several studies reporting results of 6MWD testing in patients with ATTR-CM. In a convenience sample of 78 Austrian patients with ATTR-CM, the 6MWD was 397 (119) m [15]. The mean 6MWD in the ATTR-ACT trial including 441 patients with ATTR-CM was 351 (121) m [9], and the ATTRibute-CM trial, which investigated the effect of acoramidis on ATTR-CM in 633 patients, reported a mean 6MWD of about 355 m [34, 35]. These 6MWDs are in line with findings from heart failure studies, where the average 6MWD of most cohorts was measured between 300 and 430 m [36, 37]. In our study representing “real-world” conditions, the mean 6MWD was at the lower edge of this spectrum, i.e., 310 (113) m. The mean predicted 6MWD in our study was 475 (40) m. The mean difference between the observed and predicted 6MWD was − 165 (102) m. Hence, patients with ATTRwt-CM reached a mean performance of only 65 (22) % of their age- and height-adjusted predicted ‘healthy’ walking distance. These figures impressively demonstrate the substantial functional impairment of this fragile group of patients.

The 6MWD has been used as a secondary or primary endpoint in clinical trials. Randomized controlled trials showed a significantly reduced decline in the 6MWD in patients receiving disease modifying therapy during the study period. At the end of the blinded phase of the ATTR-ACT trial, the least squares mean difference in the 6MWD between the treatment arm and placebo was 76 m in, while the respective difference was 40 m in ATTRibute-CM [34, 35]. APOLLO-B investigated the effect of the gene silencer patisiran on the 6MWD as primary endpoint in 360 patients with ATTR-CM and found a mean difference between verum and placebo group of 18 m [38]. These results imply the potential of a positive effect of TTR-targeting treatment on the 6MWD. Hence, the 6MWD can serve as useful tool to monitor disease progression and response to therapy. Nevertheless, in order to rely on the 6MWT when making clinical decisions, especially in anticipation of various potent but most likely cost-intensive therapeutic options, clinicians need to bear in mind potential confounding factors and determinants of the 6MWD.

We identified four echocardiographic parameters significantly associated with the 6MWD, when adjusted for age and height: hepatic vein dilation, mitral valve E wave velocity, TR-Pmax, and LVEF. Atrial fibrillation was another important factor: patients with atrial fibrillation walked on average 60 m less than those without atrial fibrillation. NT-proBNP, hs-Troponin T and eGFR were all significant age- and height adjusted correlates of the 6MWD. These markers are components of validated ATTR-CM disease staging systems. Higher values of cardiac enzymes and lower values of eGFR indicate an advanced disease stage associated with decreased survival rates [25, 39]. In accordance with their prognostic impact, higher NT-proBNP, higher hs-Troponin T, and lower eGFR were significantly associated with shorter 6MWD. Vice versa, a shorter 6MWD thus indicates an advanced disease stage and adds valuable information regarding a patient´s current health status. In fact, the European expert consensus on the monitoring of ATTR-CM recommends using a decline in 6MWD of 30–40 m as a marker of disease progression [40]. This is in line with the study by Ioannou et al., where the 6MWD independently predicted mortality risk across all NAC disease stages and a decline of >35 m within one year was associated with significantly increased mortality rates [17].

It is noteworthy though that besides disease progression, *congestion *should be considered as an alternative or concomitant factor determining the 6MWD. Patients with ATTR-CM are particularly prone to congestion [41], and proper diuretic management, as endorsed by heart failure guidelines of Cardiologic societies [7, 42], improves symptoms [43, 44]. A congestive state in heart failure is associated with increased natriuretic peptides as a consequence of increased intracardiac pressures and plasma dilution due to hypervolemia [45–47]. In our analysis, an improvement of 15.6 m per 1 mg/dl increment in plasma hemoglobin levels was observed, indicating higher 6MWD with hemoconcentration. Further, mitral valve E wave velocity and TR-Pmax are echocardiographic markers known to increase with higher pressures in the pulmonary circulation [45, 48]. For example, mitral E wave velocity >50 cm/s has 92% sensitivity for detecting elevated pulmonary capillary wedge pressure [45]. TR-Pmax can be used to accurately estimate pulmonary artery systolic pressure [48]. Both higher mitral valve E wave velocity and TR-Pmax were associated with lower 6MWD in our study. Similarly, dilation of hepatic veins develops with increasing congestion in heart failure [49]. Indeed, patients with hepatic vein dilation walked on average 55 m less in our study. The impact of congestion on the 6MWD is further highlighted by the fact that patients with acute decompensated heart failure walk shorter distances in 6 min than those with stable chronic heart failure [50]. Consistent with this observation, it was reported that ambulatory patients with heart failure requiring an increase in diuretic dosage subsequently presented with a decrease in 6MWD [51]. Taken together, *congestion* should be taken into consideration, when interpreting the 6MWD in clinical practice and research.

Lastly, our multivariable regression modelling demonstrated that alongside age and height, cardiovascular markers could account for up to 46% of the variability in the 6MWD, which can be considered a fairly good explanatory performance. In comparison, Felker et al. investigated the effect of NT-proBNP on the 6MWD in a post-hoc analysis of HF-ACTION, a randomized controlled clinical trial including 2331 patients with heart failure and LVEF below 35%. In a multivariable linear regression using NT-proBNP and 8 other clinical variables as predictors of the 6MWD, the obtained R² reached 30% [52]. Our results point to mitral valve E wave velocity, high-sensitivity Troponin T, atrial fibrillation and NAC disease stage as possibly robust 6MWD predictors, since they were included in all three multivariable modelling approaches (AIC based backward elimination, LASSO, clinical reasoning based regression). However, the predictive accuracy of the multivariable models was rather low. The RMSE quantifies the average difference (error) between the predicted and observed 6MWD values. Lower RMSE indicates better predictive accuracy. In our study, the RMSE was 84.14 m, 82.9 m and 85.05 m for the backward elimination, post-LASSO and clinical model, respectively. These errors are far too off given that the recommended thresholds for either ATTR-CM disease progression or increased mortality risk range between 30 and 40 m change in 6MWD [17, 40].

There are limitations to our study. Various factors beyond cardiovascular function can limit the 6MWD, such as orthopedic comorbidity, neuropathy, pulmonary or skeletal muscle disease. It is well described that ATTRwt-CM patients frequently suffer from conditions like spinal canal stenosis or arthrosis, which can contribute to decreased functional capacity [53, 54]. Systematic information about comorbidities and skeletal muscle was not available in our study, therefore, we were not able to take these possible confounders into account. Our sample size of 100 participants puts a limited statistical power on regression modelling, therefore, further research is needed to validate and strengthen our findings. The identified determinants of the 6MWD may even be mediators of adverse outcome. We were not able to elucidate that due to the lack of prospective outcome data in our cohort. Further, the conduct of the 6MWD was adjusted to the space of the premises of our outpatient clinic and a hallway of 15 m. That is lower than the 30 m length recommended by guidelines and may have introduced some bias into our measurements [8]. Nevertheless, the reference values of the normal population were also derived from the 15 m hallway [29]. Further, a 15 m hallway might be more readily available in other institutions as well, thus facilitating the implementation of serial 6MWT in the clinical routine of ATTR-CM patients.

## Conclusions

In conclusion, our study indicates that the 6MWD is related to disease severity in ATTRwt-CM, and congestion is an important confounder of the 6MWD. Cardiovascular markers explain a fair amount of 6MWD variability. Clinical and scientific interpretation of the 6MWD as well as of a potential change over time should take congestion into account.

## Supplementary Information

Supplementary material 1.

## Data Availability

The datasets generated and analyzed during the current study are not publicly available due to data protection regulations but are available from the corresponding author upon reasonable request.

## Abbreviations

6MWD: 6-minute walk distance
6MWT: 6-minute walk test
ACE: Angiotensin converting enzyme
ARB: Angiotensin receptor blocker
ARNI: Angiotensin receptor-neprilysin inhibitor
ATTRv: Amyloid-transthyretin variant
ATTRwt: Amyloid-transthyretin wildtype
BMI: Body mass index
CI: Confidence interval
CM: Cardiomyopathy
eGFR: Estimated glomerular filtration rate
hs: High-sensitivity
IVSD: Interventricular septum diameter
LASSO: Least absolute shrinkage and selection operator
LPWD: Left ventricular posterior wall diameter
LVEDV: Left ventricular end-diastolic volume
LVEF: Left ventricular ejection fraction
MAPSE: Mitral annular plane systolic excursion
MRA: Mineralocorticoid receptor antagonist
NT-proBNP: Amino-terminal pro-hormone natriuretic peptide type b
NYHA: New York heart association
RMSE: root mean squared error
TAPSE: Tricuspidal annular plane systolic excursion
TR-Pmax: Tricuspid regurgitation maximal pressure
