# Supplementary material for: Cardiovascular determinants of the 6-minute walk distance in cardiac transthyretin amyloidosis
Source: BMC Cardiovasc Disord. 2025 Nov 14;25:810. doi: 10.1186/s12872-025-05215-4 (PMC12619313; doi:10.1186/s12872-025-05215-4)
Supplement: Supplementary file 1 — Supplementary material 1. [file 12872_2025_5215_MOESM1_ESM.docx]

Supplementary Figure S1

Univariable analyses and modelling approach


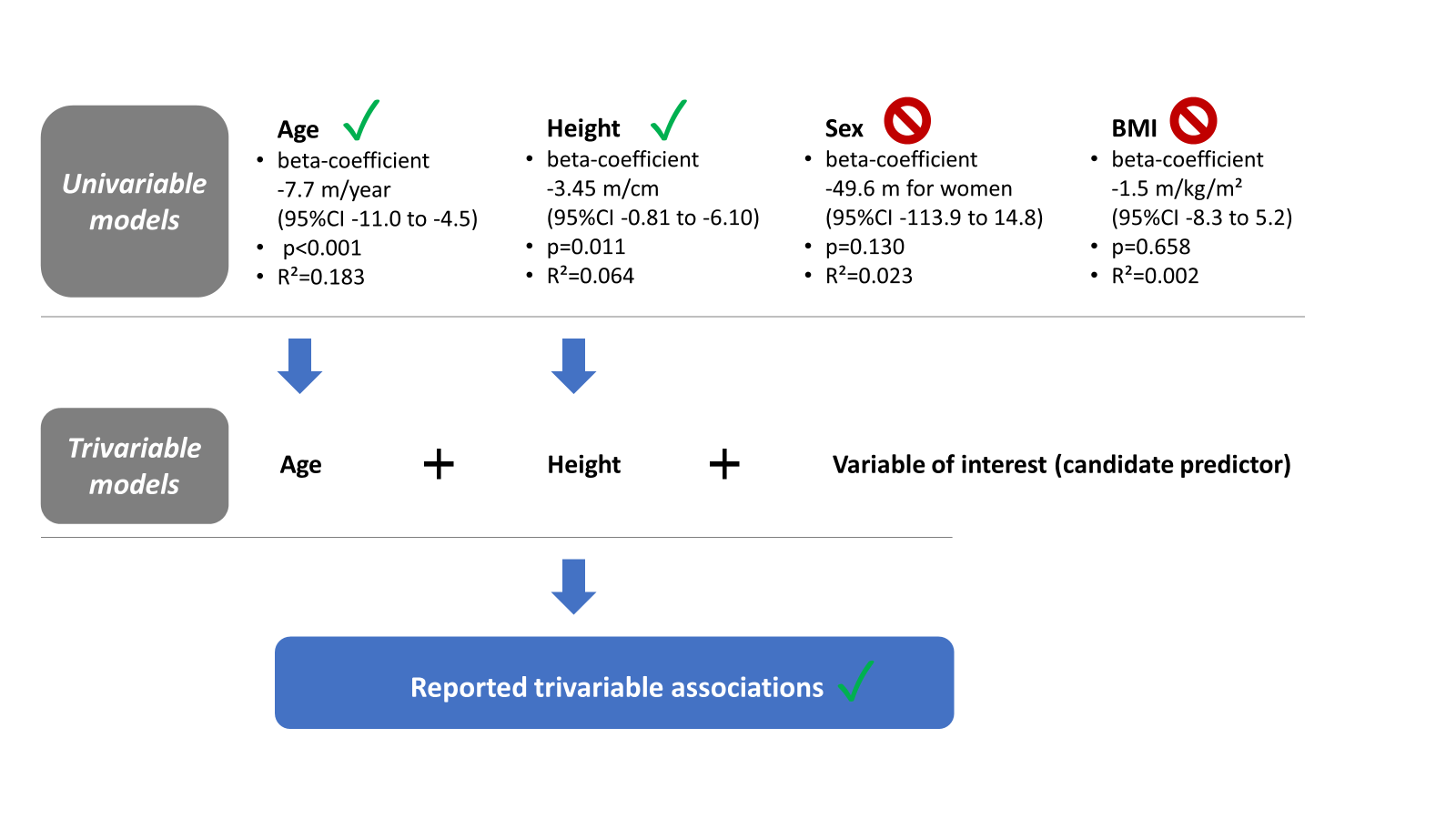


Supplementary comment to Figure S2

Clinical reasoning guided modelling approach

We aimed to build a multivariable model from significant trivariable predictors (Table 2 in main article). Age and sex were included into the clinical model as fixed covariates because we considered them important confounders, as described in the methods section of the article. We calculated step-wise linear regression models and added predictors in order of decreasing trivariable R². In detail, we inspected all calculated trivariable models for significant (p<0.05) age- and height adjusted predictors and considered these for stepwise building of a final “clinical reasoning” multivariable model with the observed 6MWD as the dependent. We started with a linear regression model including age, sex, hs-TnT and NAC stage as predictors. Hs-TnT was chosen because the trivariable hs-TNT model explained the most variance in the observed 6MWD (i.e., had the highest R²). We added NAC stage because it conveniently combines information about NT-proBNP and eGFR and we wanted to avoid collinearity by including NT-proBNP and hs-TNT into the same model. We considered adding further predictors based on decreasing R² of the trivariable models. We retained an additional predictor if it´s effect was statistically significant when added to the previous multivariable model. By this approach, the finally constructed a model including age, height, hs-TNT, NAC stage, atrial fibrillation, and MV-E wave velocity. The iterative approach is summarized in supplementary Figure S2.

Supplementary Figure S2:


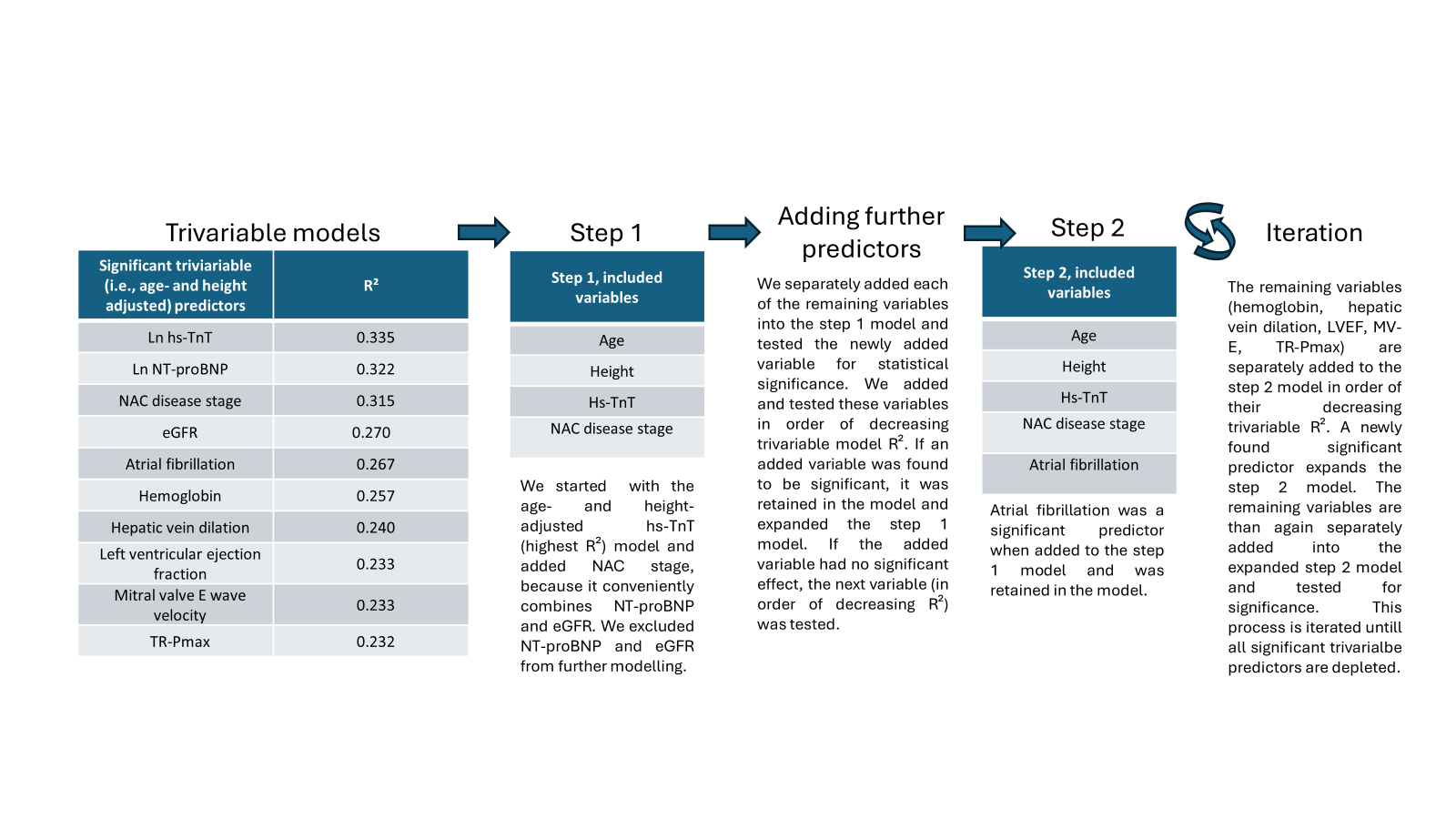


Supplementary Figure S2 legend: All depicted models refer to linear regression with the observed 6MWD as the dependent. The final clinical model is presented in the main paper. Hs: high sensitivity, Ln: natural logarithm, NAC: National Amyloidosis Center London, NT-proBNP: amino-terminal pro-hormone natriuretic peptide type B TnT: Troponin T, TR-Pmax: maximal tricuspidal regurgitation pressure grad

Supplementary Table S3:

Final LASSO linear regression model summary

| **LASSO model summary** | |
| --- | --- |
| **R²** | 0.452 |
| **RMSE (m)** | 84.52 |
| **Predictor** | **Beta** |
| Intercept | 713.38 |
| Age (years) | -5.38 |
| Height (cm) | 1.19 |
| LVEF (%) | 0.89 |
| Mitral valve E wave velocity (m/s) | -64.15 |
| TR-Pmax (mmHg) | -1.14 |
| Hepatic vein dilation (yes vs. no) | 0.00 |
| NT-proBNP (natural log-pg/ml) | 0.00 |
| High-sensitivity troponin T (natural log-pg/ml) | -35.00 |
| eGFR (ml/min/1.73m²) | 0.00 |
| Hemoglobin (g/dl) | 4.16 |
| Atrial fibrillation (yes vs. no) | -25.06 |

Supplementary Table S3 legend: Summary of final LASSO derived regression model. Note: the LASSO procedure does not provide unbiased estimates and standard errors, therefore, confidence intervals and p-values are not reported. R² and RMSE were calculated by fitting the model coefficients on the original data. eGFR: estimated glomerular filtration rate according to the CKD-EPI formula, LVEF: Left ventricular ejection fraction, NAC: National Amyloidosis Center London, NT-proBNP: amino-terminal pro-hormone natriuretic peptide type B, RMSE: root mean squared error, TR-Pmax: tricuspid regurgitation maximal pressure.
